# Seedless Hydrothermal Growth of ZnO Nanorods as a Promising Route for Flexible Tactile Sensors

**DOI:** 10.3390/nano10050977

**Published:** 2020-05-19

**Authors:** Ilaria Cesini, Magdalena Kowalczyk, Alessandro Lucantonio, Giacomo D’Alesio, Pramod Kumar, Domenico Camboni, Luca Massari, Pasqualantonio Pingue, Antonio De Simone, Alessandro Fraleoni Morgera, Calogero Maria Oddo

**Affiliations:** 1 The BioRobotics Institute, Scuola Superiore Sant’Anna, Viale Rinaldo Piaggio 34, 56025 Pontedera, Italy; alessandro.lucantonio@santannapisa.it (A.L.); giacomo.dalesio@santannapisa.it (G.D.A.); domenico.camboni@santannapisa.it (D.C.); luca.massari@santannapisa.it (L.M.); antonio.desimone@santannapisa.it (A.D.S); 2 Department of Excellence in Robotics & AI, Scuola Superiore Sant’Anna, 56127 Pisa, Italy; 3 Institute of Automation and Robotics, Poznan University of Technology, 60-965 Poznan, Poland; magdalena.mir.kowalczyk@doctorate.put.poznan.pl; 4 Department of Physics, Indian Institute of Technology Bombay, Mumbai 400076, India; pramod_k@iitb.ac.in; 5 NEST Laboratory, Scuola Normale Superiore, Piazza San Silvestro 12, 56127 Pisa, Italy; pasqualantonio.pingue@sns.it; 6 Department of Engineering and Architecture, University of Trieste, 34127 Trieste, Italy; alessandro.fraleoni@unich.it; 7 Department of Engineering and Geology, University of Chieti-Pescara, 66100 Pescara, Italy

**Keywords:** ZnO nanorods, seedless hydrothermal growth, Finite-Element Analysis, PDMS-embedded devices, tactile sensors, flexible substrates, vibrations

## Abstract

Hydrothermal growth of ZnO nanorods has been widely used for the development of tactile sensors, with the aid of ZnO seed layers, favoring the growth of dense and vertically aligned nanorods. However, seed layers represent an additional fabrication step in the sensor design. In this study, a seedless hydrothermal growth of ZnO nanorods was carried out on Au-coated Si and polyimide substrates. The effects of both the Au morphology and the growth temperature on the characteristics of the nanorods were investigated, finding that smaller Au grains produced tilted rods, while larger grains provided vertical rods. Highly dense and high-aspect-ratio nanorods with hexagonal prismatic shape were obtained at 75 °C and 85 °C, while pyramid-like rods were grown when the temperature was set to 95 °C. Finite-element simulations demonstrated that prismatic rods produce higher voltage responses than the pyramid-shaped ones. A tactile sensor, with an active area of 1 cm^2^, was fabricated on flexible polyimide substrate and embedding the nanorods forest in a polydimethylsiloxane matrix as a separation layer between the bottom and the top Au electrodes. The prototype showed clear responses upon applied loads of 2–4 N and vibrations over frequencies in the range of 20–800 Hz.

## 1. Introduction

In recent years, with the development of sensors able to measure different parameters (e.g., temperature, pressure, and humidity), mimicking the functioning of human skin receptors, tactile sensing technologies have been widely explored to reproduce the human sense of touch [1,2,3,4,5,6,7,8,9,10,11]. Such devices are mainly classified according to the transduction principle that they exploit, distinguishing between piezoresistive [12], capacitive [13], optical [9,14,15], and piezoelectric sensors [7,16,17,18].

Among the possible transduction methods, piezoelectricity has the advantage that it requires no power to generate the raw output signal, enabling the design of autonomous devices [19,20,21]. Furthermore, due to their fast response to dynamic load, piezoelectric sensors can mimic the skin fast adapting (FA) mechanoreceptors [22].

Recently, nanomaterials and nanofabrication techniques have been explored to develop tactile sensors. In particular, ZnO nanomaterials have attracted much attention in the research community due to their excellent properties, such as being self-powered, non-toxic, and highly sensitive to mechanical loads, with fast response times. Among all possible morphologies, ZnO nanorods (NRs) present enhanced mechanical properties with respect to bulk ZnO [23,24,25] and they possess an inherent permanent dipole, hence they do not require poling procedures, reducing the time and the costs of the overall fabrication process. In particular, ZnO NRs have been extensively suggested for applications as piezoelectric nanogenerators [26], and tactile sensors [7,21,27,28], in addition to other applications such as optical biosensors [29]. Several researchers investigated the electromechanical performance of ZnO NRs, finding that the electromechanical behavior of these nanostructures strongly depends on their morphological features, such as shape and aspect ratio [30,31,32].

Among the possible methods for obtaining ZnO NRs, the hydrothermal growth process is lean to perform, low-cost, and suitable for large-scale production. By tuning the growth parameters, such as temperature, time, and composition of the solution, this method allows control of the morphology of the NRs, targeting high piezoelectric responses [33,34,35]. Indeed, the hydrothermal process operates at low temperatures (<100 °C) enabling the growth of NRs on different materials, such as textiles and polymers, thus allowing the fabrication of flexible devices.

Usually ZnO NRs are grown from a ZnO thin layer (continuous or formed by nanoparticles), which acts as a seed layer (SL), i.e., nucleation site, for the subsequent hydrothermal growth of the NRs [36,37,38]. In the SL-assisted growth, the morphology of the SL (e.g., the size of the nanoparticles) deposited on the substrate plays a crucial role on the control of the alignment and the density of the ZnO NRs [39,40,41,42,43,44,45]. Specifically, on SL of small grain size, NRs growth takes place at the grain boundaries, resulting in a random alignment of the rods. On the contrary, on larger grains, the growth occurs from the grain surface, promoting the formation of vertical ZnO NRs. Annealing treatment of the SL produces larger grains, thus enabling the formation of vertical nanostructures [46,47]. Despite its effectiveness, the SL deposition step introduces an additional process that slows down the device fabrication and can lower the overall device yield.

On the other hand, it is possible to grow the NRs directly on metal electrodes such as Ag, Au, and Cu, without SL intermediation [48,49,50,51], with the metal acting as a catalyst layer for the growth [52]. In these cases, as for the SL-assisted growth, it is likely that the morphology of the metal layer affects the orientation and density of the rods. However, to the best of authors’ knowledge, there are no studies investigating this aspect, and, in absence of SL, a proper control over NRs alignment and density is usually achieved using more complex fabrication procedures, often including lithographic processes or electrochemistry [48,52,53,54]. Hence, the identification of a synthesis route involving shorter process times and enabling control of alignment, density, and morphology of the nanostructures, would be a cost-effective and time-saving solution to grow ZnO NRs.

When multiple ZnO NRs are integrated in a functional device, the piezoelectric performance of the sensor can significantly differ from that of the single sensing element, since it is affected by several manufacturing choices. In particular, the type of growth substrate, the materials constituting the conductive and the encapsulation layers, the number, and orientation of the NRs, the placement of the electrodes and the structural design of the device play a major role on the piezoresponse of the sensor and can be varied in order to enhance its performance. The optimal set of parameters that guarantees the best performance can be predicted using numerical methods, such as the Finite-Element Analysis (FEA). Experimentally, the developed sensor can be characterized on a test bench.

The most basic configuration of a ZnO NRs-based tactile sensor is the so-called sandwich configuration [33]. Typically, a flexible substrate, such as polyimide or polydimethylsiloxane (PDMS) is coated with a metal (Au, Cu, Ni etc.) that acts as bottom electrode. Sputtering, physical vapor deposition and photolithography techniques are usually employed to create a patterned conductive layer to obtain multiple arrays of sensing cells. A SL is then deposited on the metal contact and the NRs are grown via the hydrothermal method, vertically oriented with respect to the substrate. A polymer, such as SU-8 [55], Polymethyl methacrylate (PMMA) [27,56], PDMS [57], polyimide [58], or epoxy resin [59], is spin coated on the sample to encapsulate the NRs, acting as a separation layer between the electrodes and providing mechanical support. The spin-coating parameters can be adjusted to match the thickness of the polymeric layer with the length of the NRs. Then, Oxygen plasma etching can be performed to expose the NRs tips for electrical contact. Finally, another metal is deposited on top of the encapsulated NRs as upper electrode. In this configuration, the pressure applied to the sensor causes the axial compression of the NRs, resulting in voltage generation at their extremities. The piezoelectric system produces two signals that are useful for sensing: one related to the application of the pressure, the other to the release of the load. The magnitude of the voltage signal provides information about the intensity of the applied pressure, while the time lag between the initial touch and the release allows deduction of whether the device is still stressed, or the load has been removed.

Following this approach, many researchers developed arrays of ZnO NRs sandwiched between two electrodes and able to convert the applied mechanical load into a measurable voltage [4,27,60]. Other studies investigated the dynamic responses of ZnO NRs to repeated pressure loadings, for energy harvesting from natural resources such as wind, body movement, vibrations and sounds [61,62,63]. However, all these studies exploited a SL-assisted process to grow ZnO NRs.

The present work reports on a simple seedless growth of ZnO NRs onto Au electrodes, with no use of lithographic or electrochemical processes. The effect of the growth substrate topography on the density and orientation of the grown rods was investigated. The ZnO NRs were grown on Au-sputtered rigid Si wafers before and after annealing of the substrate. Indeed, due to the surface smoothness of the polished Si wafers (Root Mean Square, RMS in the order of a few tenths of nm) and their tolerance to a wide range of temperatures (melting point = 1414 °C), it is possible to assume that the roughness of the Au-deposited Si substrate is mainly given by the gold layer, enabling both to investigate the sole effect of the Au annealing on the ZnO NRs, and to collect more accurate and reproducible results. Systematic growth runs were then performed at different temperatures on annealed substrates, to identify the most effective temperature for achieving high-aspect-ratio NRs. Finite elements simulations were carried out to find the most suitable NR morphology to be adopted to integrate the as-grown NRs in a functional device. Following the results collected with rigid substrates and numerical simulations, the viability of the seedless growth of ZnO NRs was demonstrated by realizing a tactile sensor on a flexible film, with an active area of 1 cm^2^. The NRs were grown on a polyimide substrate and the NRs forest was embedded in PDMS as a separation layer between the bottom and the top Au electrodes. The device was tested under compression loads and vibrations to analyze the effectiveness of the seedless sensor in detecting mechanical loads.

## 2. Materials and Methods 

### 2.1. Hydrothermal Growth of ZnO Nanorods and Procedures of Microscopy Analyses

The ZnO NRs were grown by seedless hydrothermal synthesis on Si substrate. The samples were cleaned by following a standard procedure [64]. Then, 10 nm of titanium (Ti) and 90 nm of gold (Au) were deposited on the substrate by DC magnetron sputtering (Sputtering Sistec, Italy, model DCC 150). The Au layer was used as a catalyst to assist the growth of ZnO NRs and as an electrode for the charge carrier collection, while Ti acted as an adhesion and buffer layer to decrease the lattice mismatch between Si (100) and Au (111). Before starting the growth process, some of the Si substrates were annealed on a hotplate at 300 °C for 15 min. The topography of untreated and annealed substrates was analyzed by an Atomic Force Microscope (AFM, Veeco Innova Scanning Probe Microscope, Veeco Instruments Inc., Santa Barbara, CA, USA). The growth solution was prepared in a beaker with 60 mmol of zinc nitrate hexahydrate (Zn(NO_3_)_2_·6H_2_O, 98%, ACROS Organics™, Fair Lawn, NJ, USA) and 60 mmol (1:1 ratio) of hexamethylenetetramine (HMTA, 99%, ACROS Organics™, Fair Lawn, NJ, USA) in 100 mL of deionized water (obtained using the Elix^®^ Advantage 10 Water Purification System, Merck KGaA, Darmstadt, Germany). Subsequently, the substrate was put face-down in the solution. The beaker was covered with aluminum foil, wrapped in Teflon tape, and baked in the oven for 10 h. To observe the effect of the substrate morphology on the characteristics of the NRs, ZnO NRs growth was carried out on untreated and annealed substrates under the same experimental conditions, keeping the growth temperature constant at 85 °C. Then, different growth runs were carried out on annealed substrates to investigate the effect of the temperature (65 °C, 75 °C, 85 °C and 95 °C) on NRs morphology. As-grown ZnO NRs were observed by using the Scanning Electron Microscope (SEM, Zeiss EVO MA10, Oberkochen, Germany) and by AFM (ICON System by Bruker, Santa Barbara, CA, USA) also working in Peak Force Tapping [65] and Peak Force Piezoelectric Force Microscopy [66,67] to have information about their topographic and piezoelectric characteristics. The ZnO NRs piezoresponse was analyzed in the vertical direction via lock-in detection with the application of an alternating current (AC) voltage of 5 V at 25 kHz frequency applied to the tip. A Pt coated silicon cantilevered tip, with an elastic constant k = 2.8 N/m and resonance frequency of 80 kHz, was employed for these measurements.

The length and diameter of the pillars were measured using an open source image processing program (ImageJ, National institutes of Health, Bethesda, MD, USA), to calculate the aspect ratio. The density of the rods was estimated using ImageJ Fiji. AFM images were processed by Gwyddion software (Czech Metrology Institute, Brno, Czech Republic).

### 2.2. Pressure Sensor Fabrication and Testing 

**Figure 1:**
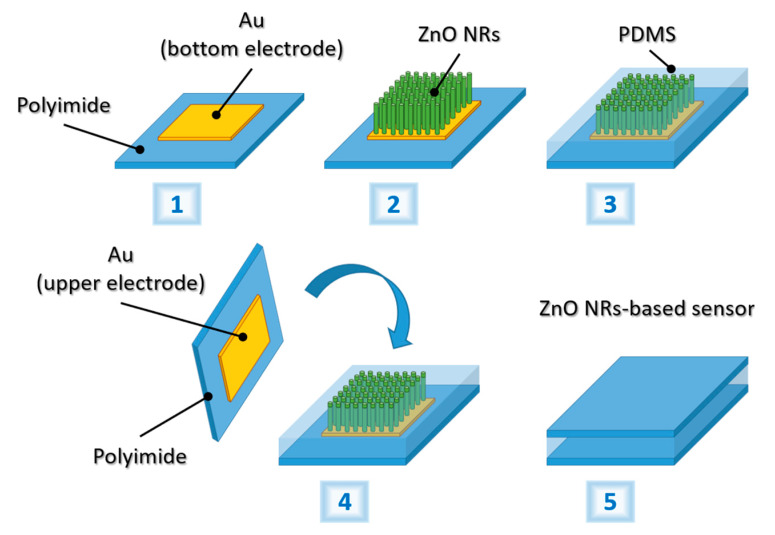
Schematics of the growth of ZnO NRs via seedless hydrothermal process on Au-coated polyimide substrates and fabrication of the pressure sensor.

The ZnO NRs were grown on a polyimide film (50 µm-thick Kapton^®^ HN from DuPont™, Wilmington, DE, USA) at 85 °C following the procedure described in Section 2.1, with no deposition of Ti. Prior to hydrothermal growth some of the substrates were annealed at 100 °C for 15 min, which was found to be the optimal temperature for the polyimide, as it is sufficiently far from the melting point of the material (400 °C). The topography of untreated and annealed substrates was analyzed by AFM. Then, the nanostructures grown on the annealed substrate were sandwiched between Au electrodes to develop a tactile sensor. The fabrication steps of the ZnO NRs-based tactile sensor from the NRs growth to their integration in a functional device are reported in Figure 1. A PDMS (Dow Corning, SYLGARD™ 184 Silicone Elastomer, Midland, MI, USA) solution was deposed on the pillars and the system was spin coated to ensure proper electrical separation between the bottom and the top electrode and provide mechanical support. PDMS was selected as it possesses ideal properties (i.e., nontoxicity, biocompatibility, flexibility, elasticity, and durability) for biomedical applications [68]. Moreover, it is not piezoelectric, hence it avoids interfering with the piezoelectric response of the ZnO NRs, enabling a clear assessment of the performance of the nanostructures. The formulation of the polymer solution and the spin-coating parameters have been optimized to adapt the thickness of the encapsulating layer to the height of the ZnO NRs. To determine the optimal PDMS layer thickness, different solutions of the polymer were deposed on glass samples at varying speeds and times, by setting the parameters of the spin-coater (Polos 200 Advanced NPP). The thickness of the final polymeric membranes was measured using an optical profilometer (DCM 3D, Leica Microsystems, Wetzlar, Germany). To complete the sensor design, an Au-coated polyimide film was placed on top of the NRs tips emerging from the PDMS, as a top electrode. Copper wires were soldered to the metal electrodes, using a low-temperature soldering wire (In/Sn), to create electrical contacts.

**Figure 2:**
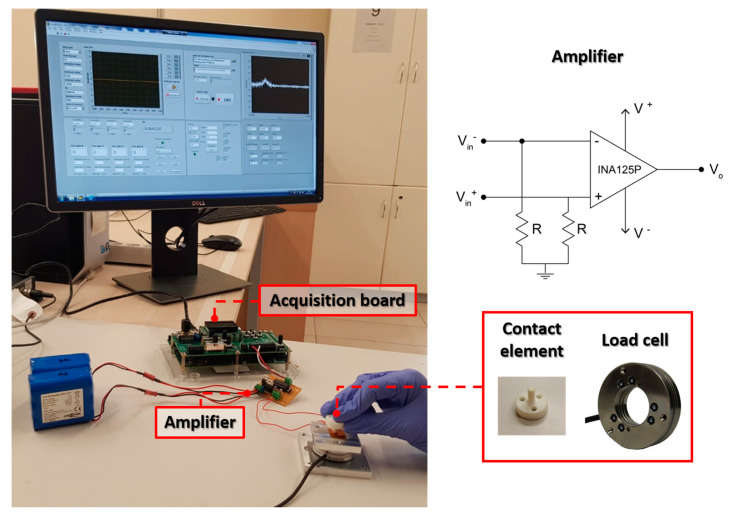
Experimental setup for sensor data acquisition during pressure-sensing (and vibration) test.

The prototype of tactile sensor based on seedless growth of ZnO NRs was tested to analyze the electromechanical response under different load conditions. The experimental setup for data acquisition is shown in Figure 2. The device was connected to a high accuracy instrumentation amplifier (INA125P, Burr-Brown^®^, Tucson, AZ, USA) with a gain of 4 (Figure 2) and the output signals were acquired at a sampling rate of 20 kHz, using an electronic board (sbRIO-9636, National Instruments^TM^, Austin, TX, USA). Moreover, the device was attached to an aluminum interface, mechanically linked to a six-axis load cell (Nano 43, ATI Industrial Automation, Apex, NC, USA) used to measure the exerted forces. Two experimental sessions were carried out to (i) investigate the voltage response to different forces applied manually by means of a 3D-printed Thermoplastic polyurethane (TPU) cylinder with a diameter of 4 mm as contact element; (ii) analyze the sensor output in response to vibrations delivered using a piezoelectric (PZT) actuator (7BB-20-6L0, MuRata, Kyoto, Japan) and modulated in frequency in the range of 20–800 Hz, while keeping constant the vibration amplitude at 150 Vpp. Such vibration parameters have been selected according to human perception performance identified in previous studies [69].

The PZT was actuated by means of a piezo haptic driver (DRV2667 Evaluation module, Texas Instruments Incorporated, Dallas, TX, USA). During the experiment, the force applied through the contact element or exerted by the PZT was acquired in real time by the load cell and then correlated with the sensor output. A graphical user interface (GUI) developed in LabVIEW (National Instruments^TM^, Austin, TX, USA) allowed to control the acquisition, to visualize data in real time, to synchronize data coming from the load cell and the device, namely the applied forces and the voltages, and to save data. Prior to each acquisition, sensor top and bottom electrodes were short-circuited to remove possible electric charges stored in the device.

#### 2.2.1. Pressure-Sensing Test

The experimental setup used in the pressure-sensing test is shown in Figure 2. The operator manually touched different points of the device surface using the TPU cylinder as contact element. The force and voltage output acquired during the experiments were analyzed in Matlab^®^ (MathWorks, Natick, MA, USA). 

#### 2.2.2. Vibration Test

The experimental setup used to generate stimuli in the *vibration test* is described in [70]. The sample was placed on the load cell and wired to the amplifier stage. Through the GUI the operator activated the PZT actuator and tuned the vibration frequency, while keeping constant the amplitude of the signal and thus the force transmitted to the sensor. Force and voltage output were analyzed in Matlab^®^ to ensure that the force did not change during the experiment and to look for possible effects on the electrical response due to the sole frequency modulation. 

### 2.3. Finite-Element Simulations 

Finite-element simulations were performed using COMSOL Multiphysics^®^ (COMSOL Inc., Palo Alto, CA, USA) to analyze the effect of (i) NRs morphology and (ii) the configuration of the Au upper electrode on NRs electromechanical performance. A single NR was modeled as a piezoelectric material with growth along the c-axis direction, by selecting ZnO (solid, c-axis) as constituent material from the COMSOL library. The piezoelectric parameters were defined as in [71].

In the simulations, pressure was applied on the top of the NR, while the bottom surface was assumed to be fixed and electrically grounded. In the simulations with Au electrode, the elasticity of the Au layer was neglected, and a floating potential was assumed for the electrostatic boundary condition (the total normal flux of the electric displacement equals the total surface charge initially present on the electrode). All the simulations were carried out in stationary conditions.

## 3. Results and Discussion

The effects of growth parameters on ZnO NRs aspect ratio, orientation, and density were investigated on rigid Si substrates. Finite-element simulations were carried out to provide useful guidelines for the design of the ZnO NRs-based tactile sensors with enhanced electromechanical performance. Based on the results collected on Si and the outcomes of the simulations, the seedless hydrothermal process was tuned to grow ZnO NRs on polyimide foils to develop a flexible ZnO NRs-based tactile sensor. The performed analysis and the fabrication and testing of the final flexible device are reported in the following sections.

### 3.1. Seedless Growth on Substrates of Different Morphologies Affects Nanorods Characteristics

To evaluate the effect of the gold grains size on the characteristics of the grown nanostructures, seedless hydrothermal growth of ZnO NRs was carried out at 85 °C comparing the results obtained on annealed and untreated Au-coated Si substrates.

**Figure 3:**
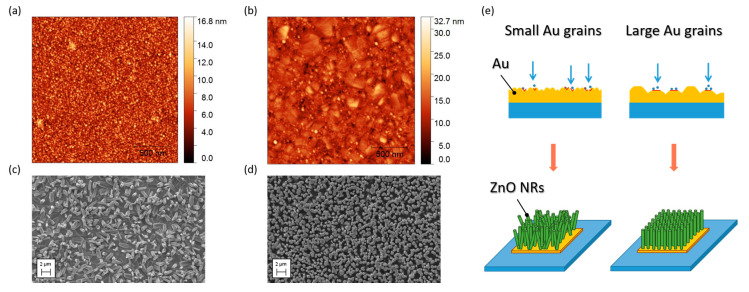
AFM images of Au deposited on Si substrates (**a**) before and (**b**) after annealing at 300 °C for 15 min. SEM images of ZnO NRs grown on substrates, (**c**) untreated and (**d**) annealed at 300 °C for 15 min. (**e**) Schematic representation of NRs nucleation and growth mechanisms occurring predominantly from the boundaries of small Au grains, and from the surfaces of large Au grains.

The AFM analysis of the substrates allowed to explore the effect of the annealing procedure on the morphology of the Au layer. Upon this treatment, the small grainy structures obtained from the as-deposited Au (Figure 3a), evolved into larger grains (Figure 3b), and the surface roughness of the Au layer increased from a RMS value of 0.5 nm to 1.1 nm, before and after annealing. The average grain size disc radius was equal to 2.5 nm and 4.7 nm for the untreated and annealed substrates, respectively. 

The different Au layer morphologies impacted the ZnO NRs characteristics: sparse and randomly oriented NRs grew on the untreated substrate (Figure 3c), presenting smaller grains, while highly dense (about 2 NRs/µm^2^) and vertically aligned NRs grew onto the annealed substrates (Figure 3d), which had much larger grains. Therefore, the size of the Au grains appears to affect appreciably the orientation of the rods. 

When looking at the SL-assisted growth, it is interesting to notice that an influence of the grain size of the ZnO SL over the morphology and the density of the hydrothermally grown NRs was observed by several researchers [46,47]. A known tool to control the grains size is the annealing treatment [39,40], which promotes the migration of the grain boundaries and the coalescence of grains [72]. In particular, on SL characterized by small grains, it was observed that NRs nucleation takes place mainly at the grain boundaries of the ZnO polycrystalline seed layer, resulting in the growth of tilted ZnO NRs with respect to the substrate plane. This is possibly due to the disordered atomic arrangement of the grain boundaries. In contrast, on larger grains the nucleation of the rods occurs from the grain surface, promoting the formation of vertical ZnO NRs. This effect has been attributed to the higher atomic arrangement order of the larger grains, which allows a more homogeneous growth [41,73]. Due to the aforementioned experimental evidence for the seedless growth on Au, it is possible to assume that the same mechanism described for the SL-assisted growth is operative also on Au-coated substrates, with smaller Au grains favoring the growth of randomly aligned ZnO NRs mainly from the Au grains boundaries, and larger grains promoting the nucleation and growth of vertical rods predominantly from the Au grains surface, as schematically reported in Figure 3e (interpretation of present experimental results based on the findings shown in [41,73]).

While other researchers reported about the seedless growth of ZnO NRs on bare Au substrates, to the best of the authors’ knowledge the present work is the first one in which an effect of the morphology (grain size) of the metal layer over the verticality and density of hydrothermally grown ZnO NRs has been reported. This finding implies that it is possible to produce ZnO NRs-based pressure-sensing devices without the need for the SL deposition step, thus simplifying the fabrication process and lowering the risk of producing defective devices. Moreover, the thermal annealing treatment of the Au layer resulted in a very uniform growth, in terms of aspect ratio and density, over surfaces as large as 1 cm^2^, which is a good starting point in view of a mass fabrication of pressure-sensitive devices.

Beside the analysis of the growth substrate morphology, the topographic and spectroscopic capabilities of the AFM technique were exploited to have information on the ZnO NRs geometry and their piezoelectricity. More in details, AFM working in Peak Force Tapping mode [65] was employed to obtain both standard topography signal and piezoelectric force microscope (PFM) response [66,67] on the same map.

**Figure 4:**
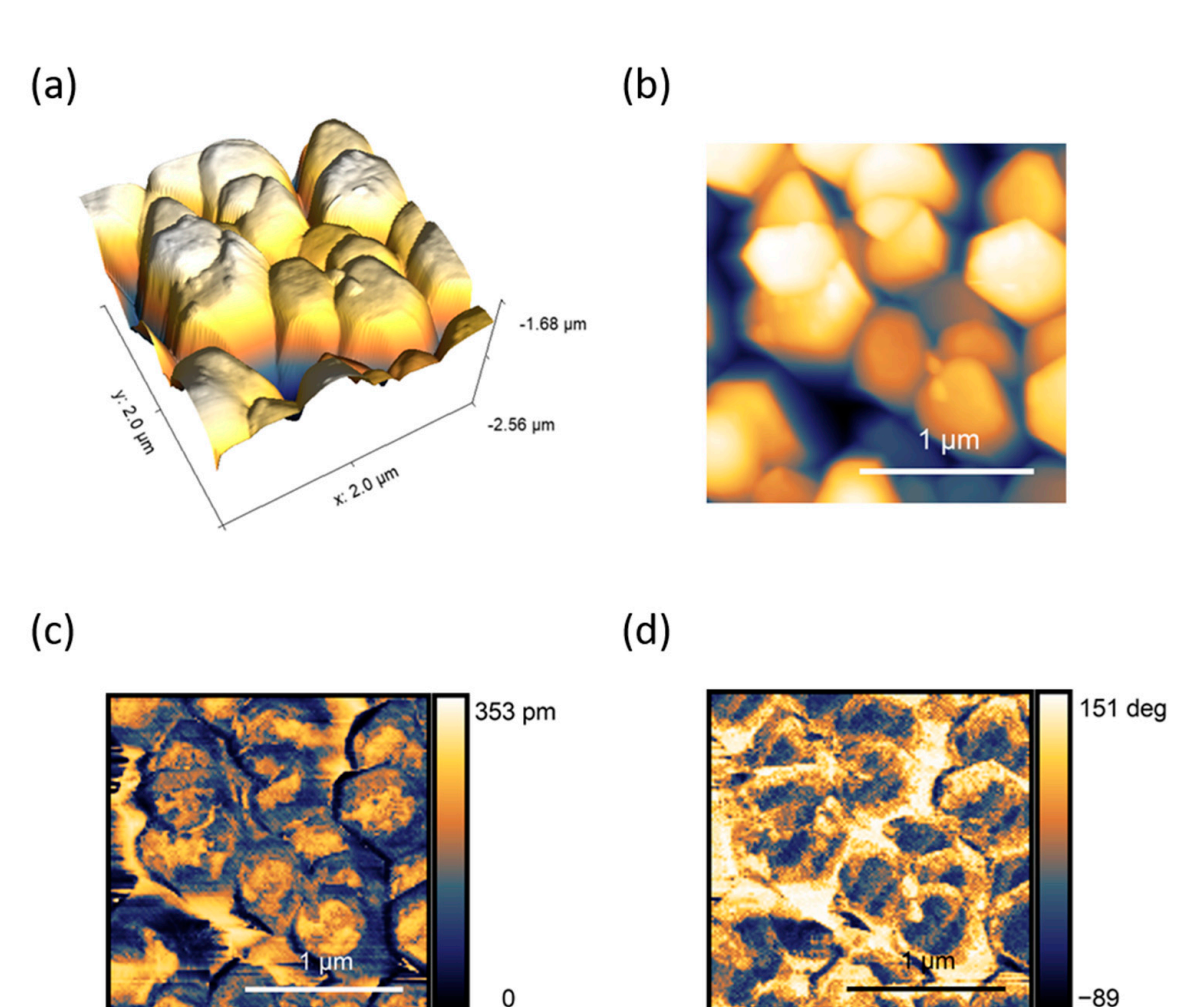
SPM images of seedless grown ZnO NRs on annealed Au-coated Si substrate. (**a**) 3D rendering of the top surface of the NRs; (**b**) Topographic false color map of the same region; corresponding PFM signal in (**c**) Amplitude and (**d**) Phase.

The images resulting from the analysis are reported in Figure 4, showing (i) the hexagonal structure of the NRs top heads (Figure 4a,b), (ii) the qualitative piezoresponse of the rods together with their local non-homogeneities (Figure 4c), and (iii) the corresponding Phase signal (Figure 4d), related to the 5 V sinusoidal waveform at 25 kHz applied between the conductive probe and the sample during the lock-in detection. The rods were able to locally interact with the AFM tip provided with the voltage signal (PFM mode), as visible from the Amplitude and Phase graphs. The PFM analysis demonstrated the presence of piezo domains also on the same rod (Figure 4c,d), nevertheless, overall the piezoelectricity of the NRs resulted to be uniform on a larger scale. 

### 3.2. Effect of Growth Temperature on the Morphology of ZnO Nanorods 

**Figure 5:**
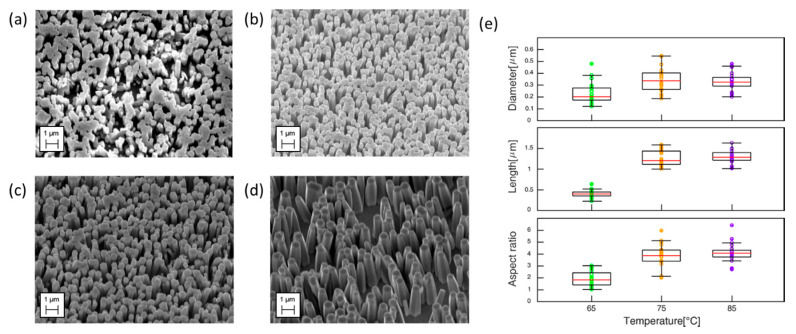
SEM images of seedless grown ZnO nanorods on annealed Au-coated Si substrate at four different temperatures (**a**) 65 °C, (**b**) 75 °C, (**c**) 85 °C and (**d**) 95 °C. (**e**) Boxplots displaying median values and interquartile ranges of diameter, length and aspect ratio of the rods grown at 65 °C, 75 °C and 85 °C.

To evaluate the effect of the growth temperature on the morphology of ZnO NRs, the characteristics of the rods that were grown at 65 °C, 75 °C, 85 °C and 95 °C were compared. Four different seedless syntheses were carried out on annealed Au-coated Si substrates, at each one of the selected temperatures, while keeping fixed all the other parameters (concentration, substrate morphology, incubation time). As reported in Figure 5a–c, the growth at 65 °C, 75 °C, 85 °C, produced vertical hexagonal prismatic rods, while pyramidal structures were observed at 95 °C (Figure 5d). The highest densities were reached at 75 °C and 85 °C (2 NRs/µm^2^). The diameter of the nanostructures showed a slight increase when moving from 65 °C to higher temperature (Figure 5e). On the contrary, the length significantly increased at higher temperatures (75–85 °C) and the resulting aspect ratio (length/diameter) was found to be much higher around 75–85 °C than at 65 °C (Figure 5e). Other researchers reported a similar trend, observing higher aspect ratios with the increase of temperature from 60 to 70 °C and pyramid-like structures when moving up to 95 °C [64]. The temperature-dependent variation of NRs aspect ratio and shape has been attributed to the relative growth rates of the different crystal planes [53]. Overall, in the present study, the increase of temperature within a given interval resulted in a higher aspect ratio of the ZnO NRs. The phenomenon is assumed to be an effect of the stronger influence exerted by the high temperatures over the growth rate of the prism top polar plane (0001) rather than over that of the lateral one [74]. When temperature is further increased up to 95 °C, both the axial and lateral growth rates of the nanostructures greatly increase, and the availability of Zn^2+^ in solution gradually diminishes, leading to the formation of NRs with progressively smaller cross-section areas (i.e., pyramid-like NRs) [64].

### 3.3. Finite-Element Simulations 

The analysis of the effect of growth parameters on NRs morphology demonstrated the possibility to obtain pyramid-like structures by increasing the temperature. To develop NRs-based tactile sensors with high voltage response it is crucial to select a morphology that maximizes the electrical output, under a given applied mechanical load. 

**Figure 6:**
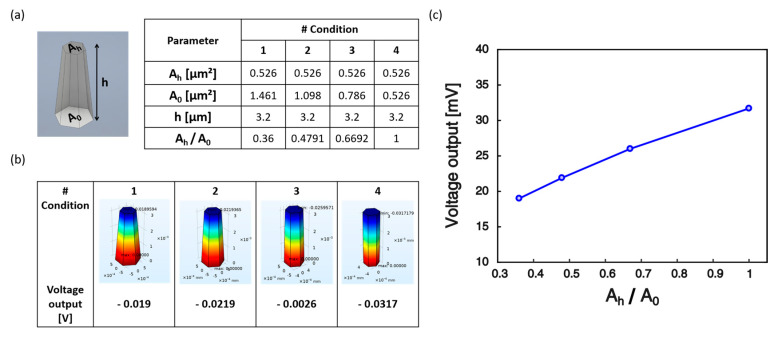
Effect of variable cross-section area. (**a**) Various geometries have been tested numerically corresponding to different area ratios ($A_{h}/A_{0}$). (**b**) Electric potential generated under pressure of 90 kPa applied on top of the NR. (**c**) The voltage output (absolute value) of the numerical model increases as the NR morphology changes from pyramid-like to hexagonal prismatic.

Finite-element simulations were carried out using COMSOL Multiphysics^®^ to analyze the effect of the morphology of ZnO NRs on their electromechanical performance. Specifically, the voltage response under constant pressure (p=90 kPa) applied on the top surface of a single NR was calculated in four different conditions, by fixing the height h of the nanostructure and the top cross-section area, $A_{h}$, while tuning the area ratio, i.e., the ratio between the top and the bottom ($A_{0}$) cross-section areas, (Figure 6a). 

The cross-section area was assumed to vary linearly along the z-axis, aligned with the NRs height:

(1) $$A\left(z\right)=A_{0}\left(1-\frac{z}{h}\right)+A_{h}\frac{z}{h}$$

The plot of the voltage response for different area ratios is shown in Figure 6b. Under the applied compressive force $F=-pA_{h}$, the nanostructure is subject to a variable longitudinal stress, $\sigma \left(z\right)=\frac{F}{A\left(z\right)}\;$, due to the variation of the NR cross-section area along the z direction. The general equation for computing the voltage generated across the NR under mechanical stress is derived in the following. Neglecting the presence of free volume charges, the solution of Gauss’ law of electrostatics provides a constant electric displacement field aligned along the z-axis. In the absence of electrodes and of an external circuit, the z-component of the electric displacement, $D_{3}$, vanishes everywhere, so that the z-component of the electric field, $E_{3}$, can be readily computed from the piezoelectric constitutive equation:

(2) $$D_{3}=\varepsilon E_{3}\left(z\right)+d_{33}\sigma \left(z\right)=0\to E_{3}\left(z\right)=-\frac{d_{33}}{\varepsilon }\sigma \left(z\right)$$

where $d_{33}$ and $\varepsilon =\varepsilon_{0}\varepsilon_{r}$ are the piezoelectric coupling coefficient along the z direction and the dielectric permittivity of the NR material, respectively. Since $E_{3}\left(z\right)=-\frac{dV\left(z\right)}{dz}$, where V(z) is the electrostatic potential, from Equation (2) we have:

(3) $$\frac{dV\left(z\right)}{dz}=\frac{d_{33}}{\varepsilon }\frac{F}{A\left(z\right)}$$

Then, accounting for Equation (1), the voltage generated across the rod is computed as:

(4) $$∆V=V\left(h\right)-V\left(0\right)=\frac{d_{33}}{\varepsilon }\frac{F}{∆A}h\ln\left(1+\frac{∆A}{A_{0}}\right)\;$$

where $∆A=A_{h}-A_{0}$. In the case of hexagonal prismatic rods ($A_{h}=A_{0}$), the longitudinal stress σ is constant and the voltage output can be estimated from Equation (4) as:

(5) $$\underset{∆A\to 0}{\lim}∆V=\frac{d_{33}}{\varepsilon }\frac{F}{A_{0}}h$$

Equations (4) and (5) have been also validated using the numerical model (Figure 6b).

In particular, in the simulations, in agreement with Equation (4), the electric potential decreased as the NRs morphology changed from hexagonal prismatic to pyramid-like (Figure 6c). The reduced voltage output is due to the lower stress in the nanostructure in the pyramidal case with respect to the hexagonal prismatic one. Indeed, a fixed applied force produces a progressively reduced stress upon the enlargement of the bottom cross-section.

### 3.4. ZnO Nanorods-Based Pressure Sensor: Polymeric Encapsulation

**Figure 7:**
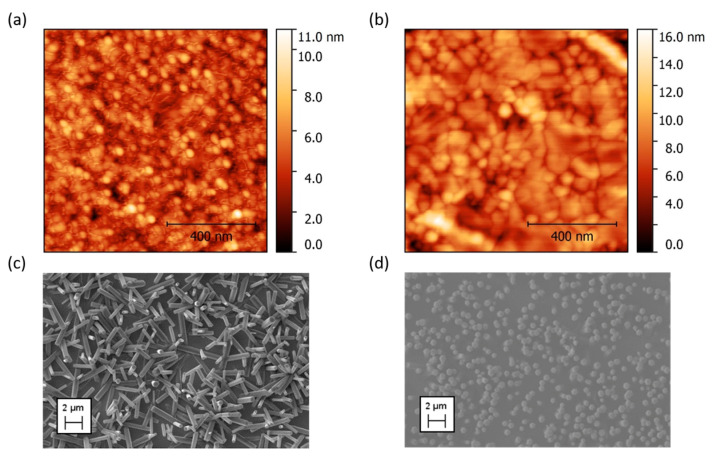
AFM images of Au deposited on polyimide substrates (**a**) before and (**b**) after annealing at 100 °C for 15 min. SEM images of ZnO NRs grown on substrates, (**c**) untreated and (**d**) annealed at 100 °C for 15 min.

The results collected on Si have been used in the development of a seedless synthesis route to grow the ZnO NRs on a flexible polyimide film. Compared to the Si wafers, the polyimide substrate possesses a higher surface roughness (RMS around 2–4 nm [75,76]) and a lower melting temperature. Nevertheless, the beneficial effects of the annealing treatment can still be exploited. Indeed, to maximize the possibility of growing vertical ZnO NRs also on these relatively rough substrates, an Au annealing was performed, even if at lower temperatures than the Si substrates. Figure 7a,b shows the AFM images of the substrate before (Figure 7a) and after (Figure 7b) the annealing at 100 °C for 15 min. Upon thermal treatment, the surface roughness of the substrate increased from an RMS value of 1.3 nm to 1.9 nm, and the average grain size disc radius increased from 2.5 nm to 3.8 nm. As shown in the SEM images (Figure 7c,d) also on polyimide the grains size appears to affect appreciably the orientation of the rods. Smaller grains resulted in sparse and tilted NRs (Figure 7c), while on the annealed substrate (Figure 7d), the rods, with an average length of 2 µm and a diameter of 600 nm, are all vertically aligned on the surface, with a good level of density (though lower than the one achieved on the Si/Au substrate, likely due to the higher roughness of the base material). Hence, the role of the morphology of the metal electrode over the final NRs orientation and density seems to be decisive for obtaining vertically oriented, reasonably dense ZnO NRs via seedless hydrothermal growth. The feasibility of annealing the substrate at relatively low temperature (100 °C) and its application to the seedless growth of ZnO NRs paves the way for the low-cost mass fabrication of flexible electronics, allowing the use, as a substrate, of polymeric materials that cannot withstand high temperature thermal treatments. 

**Figure 8:**
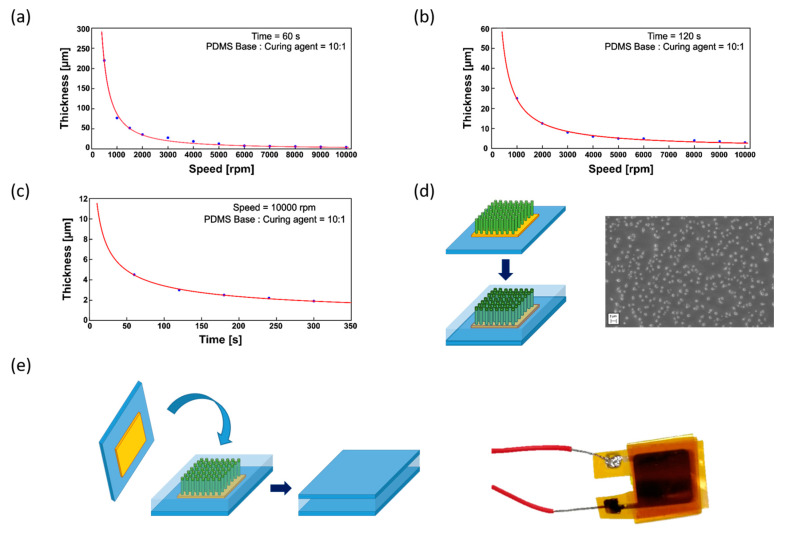
Fabrication of the ZnO-based pressure sensor from the growth of ZnO NRs on polyimide substrate. (**a**) Encapsulation of ZnO nanorods into PDMS: spinning curves of PDMS layer thickness obtained after 60 s spinning at different spinning speeds (exponential fitting *R*^2^ = 0.9951), and (**b**) 120 s (exponential fitting *R*^2^ = 0.9954). (**c**) Variation of the thickness with the spinning time at 10,000 rpm speed (exponential fitting *R*^2^ = 0.9969). (**d**) SEM image of the ZnO rods encapsulated in the polymeric matrix after dilution of PDMS in Hexane, obtained after 150 s spinning at 6000 rpm. (**e**) Final device.

The as-grown NRs (Figure 7d) were used to develop a ZnO NRs-based tactile sensor with an active area of 1 cm^2^, by means of addition of PDMS as separation layer between bottom and upper Au electrodes. To adapt the PDMS encapsulating layer to the NRs height, the thickness of the PDMS films was characterized for different spin speeds and times (Figure 8a–c) after spin-coating on glass substrate. A minimum thickness of 4.5 µm was found at a spin speed and time values of 10,000 rpm and 60 s, respectively (Figure 8a, exponential fitting *R*^2^ = 0.9951). This thickness was too high for the grown NRs, as a PDMS layer of this type would have completely coated also the top tips of the NRs, while the tips should be left free to achieve a meaningful contact with the upper electrode. The minimum height of the polymeric layer decreased to 3 µm at 10,000 rpm prolonging the spin time to 120 s (Figure 8b, exponential fitting *R*^2^ = 0.9954). A further increase of the spin time up to 300 s at 10,000 rpm spin speed allowed the achievement of a thickness of 1.9 µm (Figure 8c, exponential fitting *R*^2^ = 0.9969). However, these working parameters represented a critical condition for the spin-coater with scarce reproducibility in successive tests. To overcome this issue, PDMS was diluted in Hexane (10 wt. %) and the resulting solution was spin coated at 6000 rpm for 150 s as in [77]. After the Hexane evaporation, the resulting PDMS layer was found to have a thickness of about 1.6–1.7 µm, a good value in view of the need to have the top tips of the NRs exposed for the subsequent electrical contact with the upper electrode. Therefore, this formulation was adopted to encapsulate the ZnO NRs, and indeed the NRs tips were clearly visible after the spin-coating step (Figure 8d). Then, the fabrication of the final device was completed (Figure 8e) following the procedure described in 2.2. 

### 3.5. ZnO Nanorods-Based Pressure Sensor: Electromechanical Behavior

#### 3.5.1. Pressure-Sensing Test 

**Figure 9:**
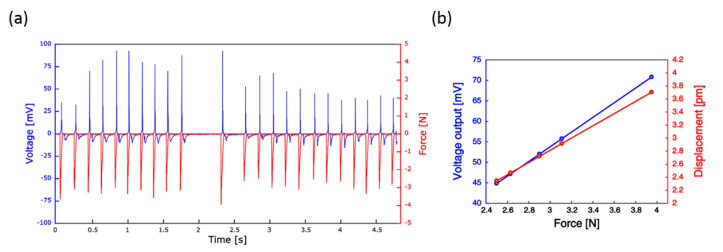
Pressure-sensing test. (**a**) Applied force (red) and sensor voltage response (blue) signals acquired during a trial. (**b**) Numerical results of displacement (red) and voltage (blue) output as function of the mechanical load applied by the operator.

Figure 9 reports the results of the pressure-sensing test. When a force was applied to the sensor by the operator, the sample showed a clear voltage response with both positive (pressing) and negative (release) peaks. The voltage signals ranged from 30 mV to 100 mV under a load of 2–4 N (Figure 9a). However, it was observed that in some cases the same applied force, as measured by the load cell, generated different voltage responses, and increases in the magnitude of the mechanical load did not always result into enhanced sensor output. To better investigate the behavior of the sensor, the model of hexagonal prismatic structure presented in Figure 6 was updated with the morphology of the nanostructures grown on polyimide film and with the values of the force applied to the device in the experiment. As demonstrated in Figure 9b, the modeled NR deformed progressively as a function of the mechanical load and a corresponding increase of the voltage output was observed, with highest value of 70 mV found at 4 N. Interestingly, under the same load condition, the real sensor output resulted to be 90 mV, which is the same order of magnitude as the numerical value. However, the simulation delivered a voltage output that increase linearly with the force, while the real device, as previously described, did not show such a clear trend. This discrepancy may be ascribed to the fact that the contact element was manually controlled by the operator, thus (i) the stimulated area over the sensor surface was not always the same and (ii) the load application speed was not kept constant, and indeed, the model described by Equation (5) did not take into account possible dependencies of NR electrical response on the strain rate. Previous studies investigated the relationship between strain rate and voltage. According to [63], the electrical response depends on both the applied load and the internal/external screening free-charge carriers that flow through the material due to the semiconductor properties of ZnO. When the nanostructure is compressed, the polarization increases and attains a maximum value, after which screening occurs. The achievement of maximum polarization and its screening are time dependent. Therefore, if the strain rate increases, the polarization reaches its maximum at a rate faster than the screening rate and the device generates higher voltages.

The strain rate dependence of the sensor output and the comparison with the voltage values obtained from the simulation (same order of magnitude) denote a piezoelectric behavior. From another point of view, the device can be thought of as a parallel plate capacitor with capacitance C, and the observed behavior could be attributed to the variations of the system capacitance upon the applied pressure. To exclude this occurrence, prior to each measurement, the sensor top and bottom electrodes have been short-circuited to remove possible electric charges stored in the device. Nonetheless, the longitudinal strain of the material and the charges generated via piezoelectric effect induce a change in the capacitance of the sensor and thus in the voltage across the NR. From the results of the simulation (Figure 9b) it is possible to quantify analytically the contribution of such a capacitive effect to the electric output of the NR. In the numerical simulation, a NR with uniform cross-section area is subject to a constant longitudinal stress σ as that given by the application of a force by the contact element. From Equation (2), the strain-induced polarization along the z direction is:

(6) $$P_{3}=D_{3}-\varepsilon_{0}E_{3}=d_{33}\frac{\sigma }{\varepsilon_{r}}$$

and the corresponding bound charge on the top surface of the NR may be evaluated as $Q=P_{3}A$, with $A=A_{h}=A_{0}$. Then, using the result in Equation (5) for the voltage output, the capacitance of the rod is given by:

(7) $$C=\frac{Q}{\left|∆V\right|}=\varepsilon_{0}\frac{A}{h}\;$$

As evident from the results of the simulations, the longitudinal strain of ZnO NRs is about 10^−6^ (Figure 9b), and from Equation (7) the relative change in capacitance due to strain-induced variations in A and h with respect to the undeformed configuration is of the same order of magnitude. Hence, in this study such an effect can be safely neglected in the evaluation of the voltage output. 

Over the tested force range (2–4 N), the tactile sensor reached its maximum load sensitivity (V/MPa) of 450 mV/MPa at 0.2 MPa (3 N). This value is in line with the performance of other ZnO NRs tactile sensors found in the literature. For example, in [27], the authors developed a tactile sensor from the encapsulation in PMMA of ZnO NRs grown on polyimide substrate, which produced an open-circuit voltage of 403 mV/MPa under external load of 0.7 MPa. The sensor presented in [60], achieved a maximum pressure sensitivity of 1.6 V/MPa, over the tested pressure range. Such remarkable performance is likely due to the lack of the encapsulating layer, which partially dissipates the applied load, hence reducing the output voltage. A further increase in the sensor sensitivity up to 90 V/MPa was reported in [78]. In the latter work, the presence of an embedding material with piezoelectric properties (Parylene C) might have contributed to the achievement of such a noticeable performance [33]. It is worth noticing that all these studies exploited a SL-assisted growth to develop ZnO NRs-based devices. On the contrary, in the presented study the sensor fabrication was achieved via simple seedless hydrothermal growth. The as-grown nanostructures have been embedded within a polymeric matrix that provides mechanical support against possible failure of the device under repeated mechanical stress, and that does not possess piezoelectric properties (hence avoiding interferences in the device performance evaluation). Moreover, the sensor has been fabricated on a flexible substrate. This evidence suggests that it is possible to obtain flexible tactile sensors based on hydrothermally grown ZnO NRs with good performance, without the extra fabrication step required for the SL deposition, thus reducing the overall development time and cost.

#### 3.5.2. Vibration Test

**Figure 10:**
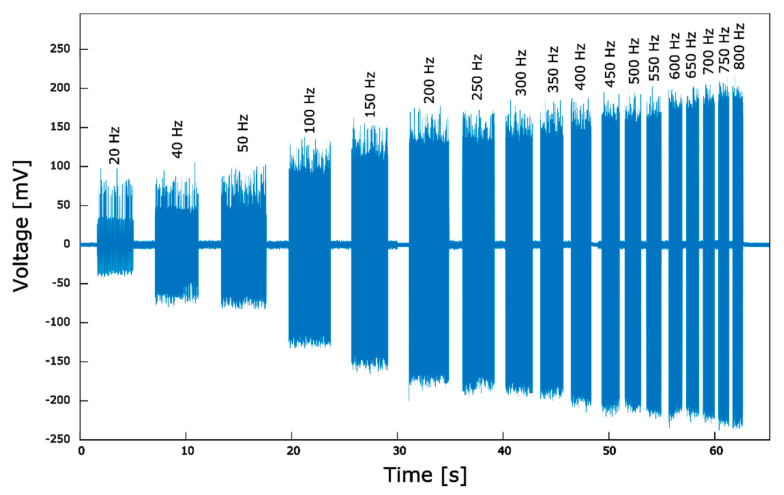
Sensor voltage response as function of the vibration frequency.

To investigate the dependence of the voltage on the material strain rate, the sensor was excited with vibrations modulated in frequency, while keeping constant the vibration amplitude (the peak-to-peak amplitude activating the PZT element was set to 150 V, following the approach described in [70]). Figure 10 shows the response of the device at different frequencies, ranging from 20 to 800 Hz, where increased frequency corresponded to increased load application rate. The sensor showed a clear response for all the frequencies tested, and the output voltage was found to increase with the frequency of the vibrations applied. As observed in [63], such a vibration frequency-dependence of the sensor output is a consequence of the relation between the polarization and screening times of the material, since the enhanced stress application speed causes the polarization to attain a higher value before it is screened.

The ability of the sensor to respond differently to a wide range of frequencies is very useful for various applications such as tactile sensor and nanogenerator, allowing detection and exploitation of vibrations from several natural resources. In fact, in human skin tactile perception is conveyed by mechanoreceptors, which behave as electromechanical transducers by translating mechanical stimuli into electrical signals to be sent to the brain. During object manipulation and surface exploration tasks, the sliding contact between the fingertips and the touched surfaces generates vibrations, which propagate through the skin and activate the mechanoreceptors. The amplitude and the frequency of such contact-induced vibrations convey information on the topographical features of the touched surface, thus determining tactile perception [79,80,81,82,83,84].

Among human skin receptors, Pacinian corpuscles present high frequency sensitivity (40-500 Hz) and are involved in the perception of fine textures [85]. At lower frequencies (<50 Hz), Meissner’s corpuscles are the mechanoreceptors responsible for vibratory sensation. They reach maximum sensitivity between 20 Hz and 50 Hz [86] and play a major role in texture discrimination [87]. Due to their fast-dynamic response to a wide range of vibration frequencies, the piezoelectric elements of the ZnO NRs-based tactile sensor proposed in this study mimic the behavior of the Pacinian and Meissner afferents and can be used to develop electronic skins for robotics applications. The use of flexible substrates and embedding layers such as polyimide and PDMS lays the ground for sensors fully conformable to non-planar rigid surfaces, hence suitable for integration into manipulators, robotic arms, and prostheses.

### 3.6. Possible Sensor Design Improvements 

The current sensor design can be improved by selecting materials that ensure mechanical integrity and stretchability.

**Figure 11:**
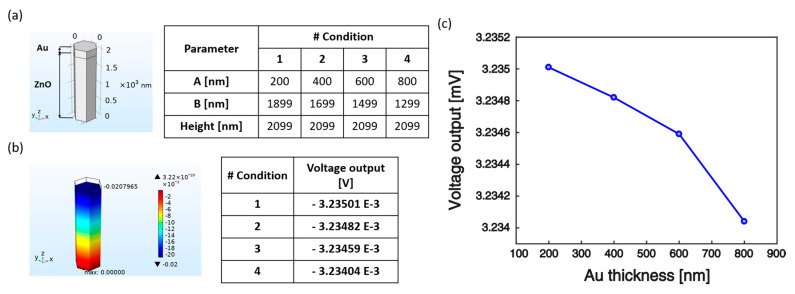
Effect of top electrode thickness on voltage response. (**a**) Four conditions have been tested numerically corresponding to different thicknesses of the Au top layer. (**b**) Electric potential generated under mechanical load of 14 kPa applied on top of the NR. (**c**) The voltage output was found to decrease as top electrode thickness increases.

Preliminary design involved the deposition of Au on top of the NRs tips emerging from the polymeric encapsulation, to ensure proper compactness of the assembly and hence facilitate its integration onto a robotics platform. However, the spin-coating of PDMS left uncovered areas over the NRs forest, causing short-circuits between the electrodes. In the future, this issue should be overcome, for example by selecting alternative materials as encapsulating layers, which can be etched via Oxygen plasma in order to smoothly adjust the thickness of the polymeric coating to adapt to the NRs height. Depending on the thickness of the encapsulating matrix, the portion of the rod exposed to Au deposition would change. In view of future device fabrication, another simulation was carried out to investigate the possible influence on the NRs piezoresponse of the variation of the amount of ZnO material covered by the top electrode. Four conditions corresponding to different thicknesses of the Au top layer (corresponding to different amounts of ZnO material exposed to Au sputtering) were tested (Figure 11a), while keeping constant the pressure exerted on top of the NR (14 kPa). Figure 11b shows the voltage generated for each condition. Specifically, the electric potential was found to slightly decrease with the increase of the top electrode thickness (Figure 11c). Indeed, for the same electric field (see Equation (2) with σ=const.) the piezopotential generated in the nanostructure is a function of the distance between the electrodes ($∆V=-E_{3}h$). The Au “cap” surrounding the NR tip creates an equipotential surface and the portion of ZnO inside the surface is an equipotential volume. Hence, the increase in thickness of the Au layer translates in reduced distance between the electrodes and thus lower voltage output. However, the effect is marginal, with variations of the voltage response in the order of a few nV.

## 4. Conclusions

The seedless hydrothermal growth of ZnO NRs was investigated as a possible route to fabricate prototypal tactile sensors on flexible substrates (polyimide), as a low-cost alternative to more complex synthesis methods.

The analysis of the surface morphology of the substrate (Au-coated Si) demonstrated that smaller Au layer grains produce tilted and sparse NRs, while larger grains, obtained from annealing treatment, allow the achievement of dense and vertical NRs. A preliminary and still qualitative PFM analysis of the as-grown ZnO NRs showed a clear piezoelectric response. 

High-aspect-ratio NRs with hexagonal prismatic shape were obtained at growth temperatures of 75 °C and 85 °C, while pyramid-like rods were grown at 95 °C. 

Finite-element simulations revealed that cylindrical rods produce higher voltage than pyramid-like structures.

On these grounds, vertically oriented, hexagonal prismatic ZnO NRs were grown on a flexible polyimide substrate and encapsulated in a PDMS matrix, then sandwiched between bottom and top Au electrodes to fabricate a ZnO NRs-based tactile sensor. The device showed a clear electrical response when tested under manually applied mechanical loads of 2–4 N, and vibrations over frequencies in the range 20–800 Hz.

The comparison between the experimental and the numerical results of the pressure-sensing test showed good agreement in terms of voltage output, while it demonstrated visible differences related to the effect of the applied load on the achieved voltage. Such differences have been attributed to three main factors, i.e., (i) the manual application of the mechanical load, (ii) the dependence of the voltage output from the NRs strain rate (in turn related to the charge screening effect deriving from the piezoresponse), which was not considered in the numerical model, and (iii) the basic settings of the model, limited to a single rod instead of multiple ones. Despite these clear limitations, the fact that the output voltage of the numerical model was of the same order of magnitude as the real one represents a good starting point for further development of the model.

In summary, this study demonstrates that it is possible to grow ZnO NRs onto flexible substrates and without any SL to attain effective tactile sensors, with the potential to cover large areas. The use of the seedless growth significantly simplifies the production process of this type of pressure sensors, eliminating a full process step. Despite these promising results, future studies will be needed for further analyzing and improving NRs structural and piezoelectric properties based on the control of the growth conditions (e.g., orientation of the Au layer crystal plane, presence of surface defects, different annealing temperatures and times, use of other metallic layers and polymeric substrates), in order to target the development of high-performance flexible tactile sensors, which could be integrated into the sensorized skins of collaborative robots and prostheses.

Future works will be focused on the optimization of the fabrication process to target the integration of ZnO NRs-based tactile sensors onto a robotic platform or their use as bio-hybrid sensors [88] or patterned substrates [89] in combination with biological cultures, and on deeper investigations over the effect of the substrate morphology and the crystallinity of the metal layer on the growth and performance of the ZnO NRs. Possible improvements to the above described sensor design could derive from selecting materials that possess stretchability coupled to mechanical resistance to fatigue and scratching.
